# Genome-wide association study of insect bite hypersensitivity in two horse populations in the Netherlands

**DOI:** 10.1186/1297-9686-44-31

**Published:** 2012-10-30

**Authors:** Anouk Schurink, Anna Wolc, Bart J Ducro, Klaas Frankena, Dorian J Garrick, Jack CM Dekkers, Johan AM van Arendonk

**Affiliations:** 1 Animal Breeding and Genomics Centre, Wageningen University, P.O. Box 338, Wageningen, 6700 AH, the Netherlands; 2 Department of Genetics and Animal Breeding, Poznan University of Life Sciences, Poznan, Poland; 3 Department of Animal Science, Center for Integrated Animal Genomics, Iowa State University, Ames, USA; 4 Quantitative Veterinary Epidemiology Group, Wageningen University, P.O. Box 338, Wageningen, 6700 AH, the Netherlands

## Abstract

**Background:**

Insect bite hypersensitivity is a common allergic disease in horse populations worldwide. Insect bite hypersensitivity is affected by both environmental and genetic factors. However, little is known about genes contributing to the genetic variance associated with insect bite hypersensitivity. Therefore, the aim of our study was to identify and quantify genomic associations with insect bite hypersensitivity in Shetland pony mares and Icelandic horses in the Netherlands.

**Methods:**

Data on 200 Shetland pony mares and 146 Icelandic horses were collected according to a matched case–control design. Cases and controls were matched on various factors (e.g. region, sire) to minimize effects of population stratification. Breed-specific genome-wide association studies were performed using 70 k single nucleotide polymorphisms genotypes. Bayesian variable selection method Bayes-C with a threshold model implemented in GenSel software was applied. A 1 Mb non-overlapping window approach that accumulated contributions of adjacent single nucleotide polymorphisms was used to identify associated genomic regions.

**Results:**

The percentage of variance explained by all single nucleotide polymorphisms was 13% in Shetland pony mares and 28% in Icelandic horses. The 20 non-overlapping windows explaining the largest percentages of genetic variance were found on nine chromosomes in Shetland pony mares and on 14 chromosomes in Icelandic horses. Overlap in identified associated genomic regions between breeds would suggest interesting candidate regions to follow-up on. Such regions common to both breeds (within 15 Mb) were found on chromosomes 3, 7, 11, 20 and 23. Positional candidate genes within 2 Mb from the associated windows were identified on chromosome 20 in both breeds. Candidate genes are within the equine lymphocyte antigen class II region, which evokes an immune response by recognizing many foreign molecules.

**Conclusions:**

The genome-wide association study identified several genomic regions associated with insect bite hypersensitivity in Shetland pony mares and Icelandic horses. On chromosome 20, associated genomic regions in both breeds were within 2 Mb from the equine lymphocyte antigen class II region. Increased knowledge on insect bite hypersensitivity associated genes will contribute to our understanding of its biology, enabling more efficient selection, therapy and prevention to decrease insect bite hypersensitivity prevalence.

## Background

Insect bite hypersensitivity (IBH) is a common allergic skin disease in various horse breeds found throughout the world, and results from bites of *Culicoides* spp. Sensitive horses develop a severe itch, which results in self-inflicted trauma and severely affected horses sometimes need to be euthanized [1]. Welfare of affected horses is therefore reduced. No cure is available, and methods to prevent or reduce clinical symptoms often require dedication from the owner and greatly differ in efficiency e.g. [1-5]. Owners of affected horses incur costs related to preventive or curative methods and veterinary consultation. Moreover, the commercial value of affected horses is reduced and use of affected horses can be restricted due to discomfort and disfiguration [1].

Insect bite hypersensitivity is a multi-factorial disorder that is affected by environmental and genetic factors. Environmental factors are, among others, related to *Culicoides* spp. density. Partial genetic control has been confirmed in various horse breeds [6-8]. Monogenic inheritance of sensitivity to IBH has been rejected by segregation analysis [9], which showed a polygenic mode of inheritance. However, little is known on the genes contributing to genetic variance. Genomic research on IBH using a candidate gene approach or genome-wide association study (GWAS) has been limited. Using a candidate gene approach, Andersson et al. [10] showed that variants within the major histocompatibility complex (MHC) class II region are associated with IBH sensitivity. The same allele (*COR112*:274) increased IBH sensitivity in both Swedish-born Icelandic horses (odds ratio = 4.19) and Exmoor ponies (odds ratio = 1.48). Moreover, homozygosity across the MHC class II region increased IBH sensitivity in both breeds. Serological research on IBH has also shown a significant difference in the distribution of specific MHC antigens between cases and controls [11,12]. The MHC genes in the horse, known as equine lymphocyte antigen (ELA) genes, are located on horse chromosome (*Equus caballus*) ECA20 and their resulting protein structures recognize many foreign molecules, thereby evoking an immune response [13].

Using GWAS, Schurink et al. [14] found associations between ECA20 and IBH. The identified genomic region was approximately 8 Mb from the MHC region and was poorly covered in single nucleotide polymorphisms (SNP) from the marker panel. Schurink et al. [14] identified 24 SNP on 12 chromosomes in Shetland pony mares associated with IBH sensitivity (− log _10_ (p) >2.5). Insect bite hypersensitivity is observed in many horse breeds throughout the world and could have common genetic components across breeds. Across-breed analyses could facilitate fine-mapping by reducing the length of associated genomic regions, since haplotypes shared across breeds are expected to be shorter than within-breed haplotypes e.g. [15,16].

The aim of our study was to expand these findings through identification and quantification of genomic associations with IBH using phenotypic and SNP information from Shetland pony mares and Icelandic horses in the Netherlands. Knowledge of genomic regions associated with IBH will contribute to our understanding of its biology, enabling more efficient selection, therapy and prevention in order to decrease IBH prevalence.

## Methods

### Animals and phenotypes

Cases were defined as individuals showing clinical IBH symptoms, while controls were free of symptoms despite exposure to *Culicoides* spp. Selection of cases and controls was described in detail by Schurink et al. [14], and cases and controls were matched on various factors to minimize effects of population stratification. Shetland pony mares were recruited through routine inspections in 2009 and through publications by the studbook in their magazine and on their website in 2010. Shetland pony mares were matched on withers height category, coat colour, location and sire. Icelandic horses were recruited in 2010 through publications on various equine related websites and were matched on coat colour, location, sex, importation from Iceland (yes/no) and sire. Age at onset is generally between 2 and 4 years-of-age e.g. [17]. Therefore, controls were required to be at least 4 years-of-age and to have been at least one year at risk for developing symptoms. Proximity to a case was required to ensure exposure to *Culicoides* spp. and thereby increase reliability of phenotypes on controls. Paternal half-sibs were sought to minimize population stratification due to pedigree. The dataset (Table 1) contained 200 Shetland pony mares and 146 Icelandic horses. The same Shetland pony mares analysed by Schurink et al. [14] were included in our study, although 70 k genotype data were available, since the mares were re-genotyped.

**Table 1:** **Distribution of characteristics** (**numbers**) **of Shetland pony mares and Icelandic horses for cases and controls**

	**Shetland pony mares**			**Icelandic horses**		
**Trait**	**Cases**	**Controls**	**Total**	**Cases**	**Controls**	**Total**
**Number of animals**	103	97	200	73	73	146
**Year of scoring**						
2009	83	78	161	-	-	-
2010	20	19	39	73	73	146
**Month of scoring**						
September	52	52	104	22	29	51
October	47	42	89	51	44	95
November	4	3	7	-	-	-
**Veterinarian**						
1	97	93	190	73	73	146
2	6	4	10	-	-	-
**Pedigree**						
Number of sires	84	86	129	57	61	95
Number of dams	100	93	187	68	67	126
**Age**, **years**						
Mean (SD)	7.1 (4.5)	8.3 (4.4)	7.7 (4.5)	13.1 (6.0)	12.6 (5.9)	12.8 (5.9)
Range	0 – 23	4 – 22	0 – 23	4 – 29	4 – 35	4 – 35
**Sex**						
Female	103	97	200	51	43	94
Male	-	-	-	22	30	52
**Withers height category**						N/A^a^
Mini	28	24	52			
Small	17	18	35			
Middle	32	27	59			
Tall	26	28	54			
**Imported from Iceland**			N/A^a^			
Yes				17	2	19
No				56	71	127
**Coat colour**						
Bay	5	4	8	11	7	18
Black	51	52	103	16	11	27
Black paint	6	4	10	2	3	5
Chestnut	26	23	49	8	14	22
Chestnut paint	8	6	14	-	2	2
Other	7	8	15	28	35	63
Silver dapple	-	-	-	8	1	9

^a^N/A = not applicable.

Participating owners were visited by an experienced veterinarian and researcher to take blood samples, score phenotypes and conduct an IBH related questionnaire (more details in Schurink et al. [14]). All Icelandic horses and most Shetland pony mares (95.0%) were scored by the same veterinarian (Table 1) to ensure uniform classification. Blood sample collection from Shetland pony mares and Icelandic horses was approved by the Board on Animal Ethics and Experiments from Wageningen University (experiments 2009055 and 2010109).

### Data

Shetland pony mare data contained 103 cases and 97 controls collected in autumn 2009 or 2010 (Table 1). Data contained half-sib mares (50.0% of the data) descending from 41 sires with both case(s) and control(s) among their offspring, and mares (50.0% of the data) descending from 88 sires with only case(s) or control(s) among their offspring. Mares were located on 73 premises. The number of mares per premise ranged from 1 (23.3% of all premises) to 9 (2.7%) and the mean number of mares per premise was 2.7.

Icelandic horse data contained 73 cases and 73 controls collected in autumn 2010 (Table 1). It contained both females (64.4%) and males (i.e. geldings and stallions; 35.6%) (Table 1). In total, 13.0% of Icelandic horses were imported from Iceland and 87.0% were born in Europe (mainly the Netherlands) (Table 1). Data contained half-sib horses (45.2% of the data) descending from 23 sires with both case(s) and control(s) among their offspring, and horses (54.8% of the data) descending from 72 sires with only case(s) or control(s) among their offspring. Horses were located on 31 premises. The number of horses per premise ranged from 1 (19.4% of all premises) to 14 (3.2%) and the mean number of horses per premise was 4.7.

### Genotyping and quality control

Genotypes from all Shetland pony mares and Icelandic horses were obtained using the equine HD chip (Illumina Inc., San Diego, CA) containing 65 157 SNP. Those SNP with a call-rate < 90% or minor allele frequency ≤ 0.02 were excluded from the data. Call-rate per animal was considered sufficient (> 90%) for all animals. The majority (319 out of 346) of animals had a call-rate greater than 99%. After breed-specific quality control (applying the same quality control to each breed separately), the Shetland pony mare data contained 46 888 SNP and the Icelandic horse data contained 51 453 SNP.

### Population stratification analysis

Cases and controls were matched on various factors to minimize effects of population stratification and thereby reduce possible spurious associations. To test whether matching of cases and controls in Shetland pony mares and Icelandic horses was successful, the relation between IBH (case or control, binary phenotype) and matching factors was assessed in univariable and multivariable models using the LOGISTIC procedure incorporated in SAS 9.2© software (SAS Institute Inc., Cary, NC). Fixed effects of withers height category, coat colour, sex, import from Iceland, veterinarian and month and year of scoring, and the covariate of age of the animal were tested for significance.

Similar genomic kinship within and across cases and controls indicates successful matching on pedigree. Breed-specific genomic kinship among animals was therefore computed using the *ibs* function of the R package GenABEL [18] as:

$$f_{i,j}=\Sigma_{k}\frac{\left(x_{i,k}-p_{k}\right)\left(x_{j,k}-p_{k}\right)}{\left(p_{k}\left(1-p_{k}\right)\right)}\text{,}$$

where *f*_*i*,*j*_ is the genomic kinship (identity-by-state) between animal *i* and *j*, based on *k* = 48 810 autosomal SNP in Icelandic horses (SNP with call-rate < 90%, monomorphic SNP and SNP on the X chromosome excluded) and 44 576 autosomal SNP in Shetland pony mares; *x*_*i*,*k*_ or *x*_*j*,*k*_ are the genotypes (coded as 0, ½, 1) of the i^th^ or j^th^ animal for SNP *k* and *p*_*k*_ is the frequency of the allele (top strand). The genomic kinship matrix was transformed to a distance matrix to perform classical multidimensional scaling [19], which returned the first two principal components. The principal components for each breed were plotted to visualize distances between animals and more specifically between cases and controls. Further, Icelandic horses were categorized into ‘imported from Iceland’ or ‘born in Europe’ to see whether their genetic background differed.

### Genome-wide association study

Breed-specific GWAS were performed using genotypes from the same marker panel but the number of SNP after quality control differed between Shetland pony mares (n = 46 888) and Icelandic horses (n = 51 453). The Bayesian variable selection method Bayes-C with a threshold model, described by Kizilkaya et al. [20] and implemented in the GenSel software ( http://bigs.ansci.iastate.edu/), was used to identify and quantify genomic regions associated with IBH. Method Bayes-C assumes a common variance for all SNP in the model and is less sensitive to the prior for genetic variance e.g. [21-23] compared to Bayes-B as described by Meuwissen et al. [24]. Method Bayes-C fits all SNP simultaneously using a mixture threshold model and assuming additive SNP effects:

$$\eta =\mu +\sum_{j=1}^{K}Z_{j}u_{j}\delta_{j}$$

where *η* is the linear predictor that is related to observed IBH phenotypes (case/control) through a probit link function and was sampled during each iteration from a normal distribution that comprises the liability scale corresponding to the observed threshold score following Sorensen et al. [25]; *μ* is an overall mean; *K* is the number of SNP; *Z*_*j*_ is the column vector representing the genotype covariate at SNP *j* (input as AA = −10, AB = 0, BB = 10 with missing genotypes set to the average value of the particular SNP in the data set); *u*_*j*_ is the random allele substitution effect of SNP *j*, and *δ*_*j*_ is a random 0/1 variable indicating the absence (with probability *π*) or presence (with probability 1 − *π*) of SNP *j* in the model.

In our analyses, *π* was set to 0.999, resulting in roughly 30 to 70 SNP being included in the model in any particular iteration. Fewer SNP than individuals were fitted in any iteration to decrease the risk of overfitting the data, and previous work [14] showed that a limited number of SNP reach significance level. The allele substitution effect for SNP *j* (*u*_*j*_) was assumed normally distributed ∼ *N*(0, *σ*_*u*_^2^) conditional on *σ*_*u*_^2^ when SNP *j* was included in the model (*δ*_*j*_ = 1), but *u*_*j*_ was 0 when *δ*_*j*_ = 0. Variance *σ*_*u*_^2^ was assumed to follow a scaled inverse chi-square distribution with *v*_*u*_ = 4 degrees of freedom and scale parameter *S*_*u*_^2^, which was derived from the additive genetic variance as $\frac{\sigma_{a}^{2}}{K\left(1-\pi \right)2\overset{―}{p}\overset{―}{q}}$ according to Gianola et al. [26] and Kizilkaya et al. [20]. The prior of *σ*_*u*_^2^ was derived from the heritability of IBH on the liability scale (= 24%), as estimated in a pedigree-based population genetic analysis by Schurink et al. [7]. Residual variance *σ*_*e*_^2^ is not identifiable and was set to 1 and not sampled. Sampling of effects is described in more detail by Kizilkaya et al. [20]. A total of 200 000 Markov chain Monte Carlo (MCMC) iterations were run, with a burn-in period of 20 000 iterations.

Model frequency, i.e. the proportion of total post burn-in iterations in which a particular SNP was included in the model, was used as evidence for an associated SNP. However, if consecutive SNP are in high linkage disequilibrium (LD) with a particular quantitative trait locus (QTL), effects and model frequencies may be distributed across those SNP, and effects and model frequencies of individual SNP will completely capture the effects of the QTL. Thus, a window approach, which accumulates effects of adjacent SNP, was used to better identify genomic regions associated with QTL [27].

The approach described by Wolc et al. [28] and implemented in version 4.0 of the GenSel software ( http://bigs.ansci.iastate.edu/) was used to identify associated windows (i.e. genomic regions). For this purpose, physical map order (build EquCab2.0) was used to allocate SNP to consecutive non-overlapping 1 Mb windows (n = 2376), and the posterior distribution of the percentage of genomic variance explained by each of these windows was derived. For this purpose, the variance of genomic breeding values for each window (= window genomic variance) was computed among individuals for every 100^th^ iteration of the MCMC chain based on the marker effects sampled in that iteration. Window genomic variance was divided by genomic variance explained (sum of all SNP) across the genome in that particular iteration to determine the percentage of genomic variance explained by the window. The resulting posterior distribution of the % variance of each window was used for testing. The posterior distribution included results from iterations that excluded the window (or SNP) from the model. Window genomic variance greater than 0.04% [i.e. the expected percentage of variance explained by each window in an infinitesimal model ( $\frac{1}{2376}\times 100$)], was used as a threshold to declare regions that explained more variance than expected.

## Results

### Phenotypes and questionnaire results

Questionnaire results from Shetland pony mare and Icelandic horse cases are shown in Table 2. In Icelandic horses, questionnaire data from 14 out of 73 cases was missing. The observed clinical symptoms of IBH in all cases are summarized in Table 3. Severity of itch was lower in Icelandic horse cases compared with Shetland pony mare cases (Table 2), probably because eczema blankets (a preventive measure) were used in many Icelandic horse cases. Preventive or curative measures were applied more often for Icelandic horse cases than for Shetland pony mare cases (Table 2) and more measures per case were applied to Icelandic horse cases compared to Shetland pony mare cases. Owners of cases replied that they experienced negative effects of IBH, as it reduces equine welfare, requires much time and limits rideability and marketability. For both Icelandic horses and Shetland pony mares, questionnaire results and observed clinical symptoms agreed with the typical course of IBH e.g. [29].

**Table 2:** **Questionnaire results** (**numbers**) **from Shetland pony mare and Icelandic horse cases of insect bite hypersensitivity**

	**Shetland pony mares**	**Icelandic horses**
**Trait**		
**Age at onset**		
Younger than 2 years	10	1
2 to 5 years	64	30
6 to 10 years	16	13
11 years or older	2	6
Unknown	11	9
**Duration IBH**		
1 year	18	-
2 years	23	-
3 years or more	57	59
Unknown	5	-
**Onset of symptoms**		
Spring	70	48
Summer	26	6
Autumn	1	-
Unknown	6	5
**Disappearance of symptoms**		
Summer	7	-
Autumn	84	53
Winter	1	1
Chronic	3	-
Unknown	8	5
**Severity of symptoms over years**		
Increases	9	1
Decreases	7	7
Remains equal	48	10
Varies	22	35
Unknown	17	6
**Severity of itch**		
Mild	26	26
Moderate	33	22
Severe	38	9
Unknown	6	2
**Preventive or curative measures**		
Yes	85	58
No	18	1
**Applied measures**^**a**^		
Eczema blankets	28	51
Local treatment with oil or cream	72	45
Insecticide	22	25
Related to nutrition	3	17
Stabling	4	5

^a^In several cases more than one preventive measure was applied; sum of applied measures per breed therefore exceeded the total number of cases per breed.

**Table 3:** Insect bite hypersensitivity symptoms on various locations in Shetland pony mare and Icelandic horse cases

	**Shetland pony mare cases**	**Icelandic horse cases**
**Clinical symptoms**		
Hair loss	100	71
Thickening of skin	89	71
Crusting	26	17
Scaling	9	4
Open wounds	9	1
**Affected location**		
Crest	102	72
Base of the tail	86	61
Hindquarters	22	-
Head	4	12
Abdomen	1	8
Other	4	-

Clinical symptoms were scored by an experienced veterinarian and are presented as the number of cases with this particular clinical symptom; in total, 103 Shetland pony mare cases and 73 Icelandic horse cases were scored.

### Population stratification analysis

Matching of Shetland pony mares to minimize effects of population stratification was successful, as none of the matching factors had a significant effect on IBH (p < 0.05). Analysis of matching factors in Icelandic horses indicated that import from Iceland (p = 0.002) had a significant effect on IBH.

**Figure 1:**
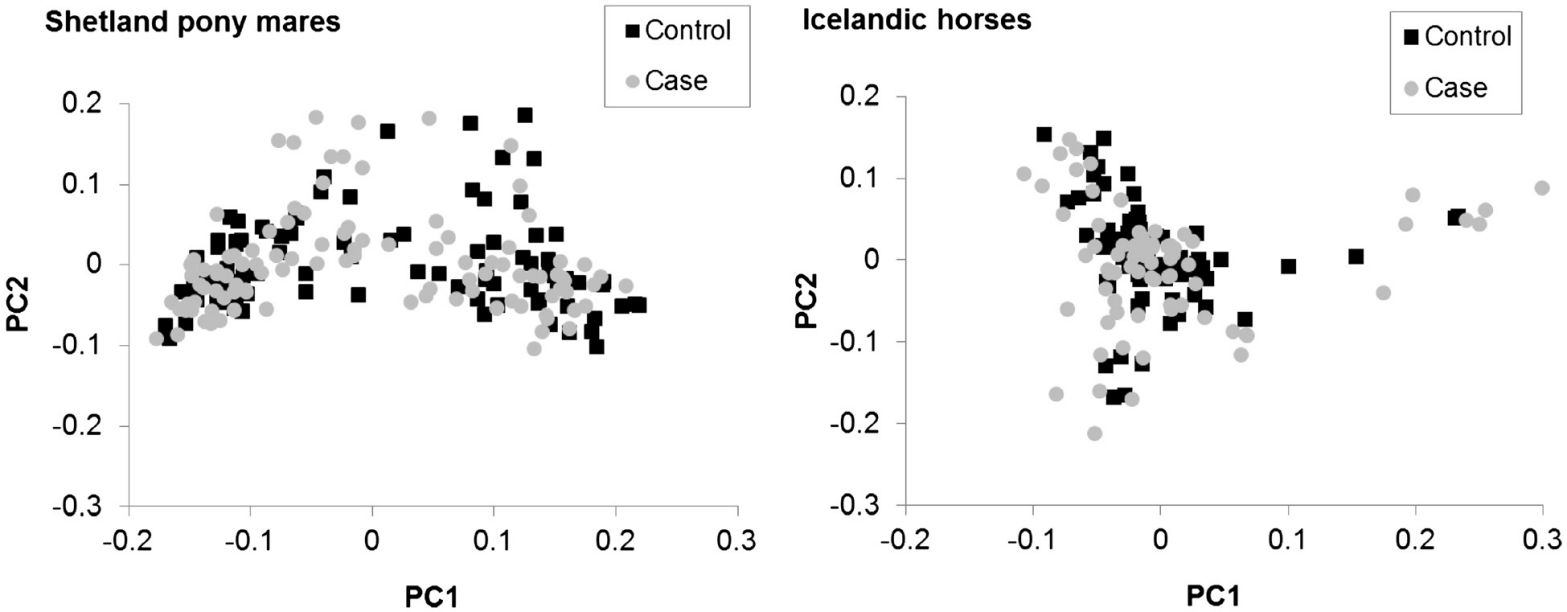
**Multi**-**dimensional scaling plots of the genetic distance between animals in Shetland pony mares and Icelandic horses**. Each point corresponds to one animal and indicates the distance between animals represented by the first two principal components (PC1 and PC2), based on the genomic kinship matrices.

**Figure 2:**
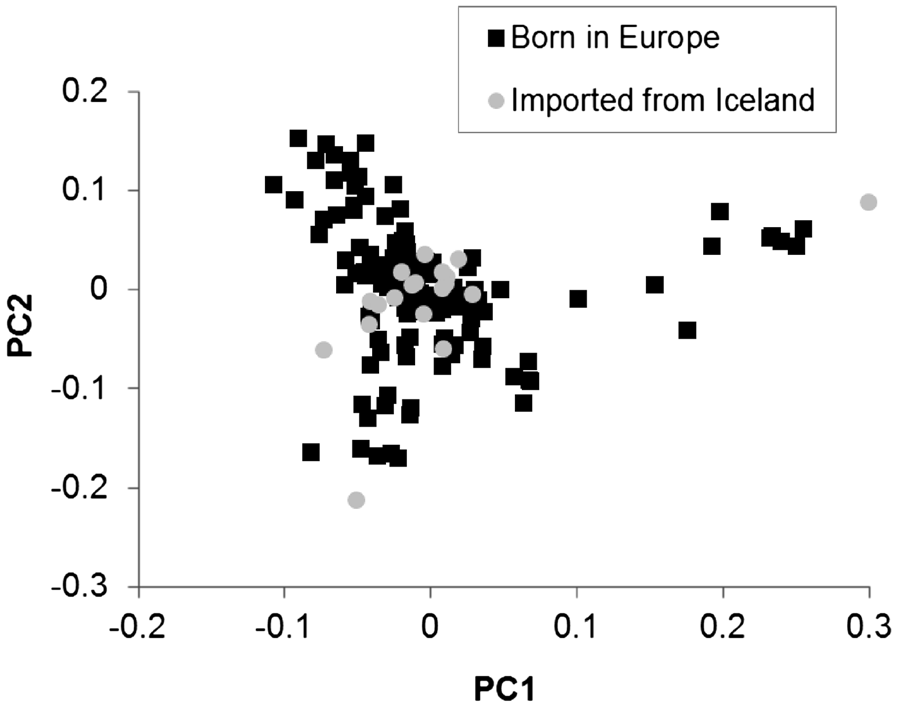
**Multi**-**dimensional scaling plot of the genetic distance between imported Icelandic horses and Icelandic horses born in Europe**. Each point corresponds to one animal and indicates the distance between animals represented by the first two principal components (PC1 and PC2), based on the genomic kinship matrices.

To test whether matching of cases and controls based on sire was successful, breed-specific genomic kinship among animals was computed based on identity-by-state of SNP genotypes. Figures 1 and 2 show the first two principal components of the transformed breed-specific kinship matrices to visualize genetic distances between animals. The multidimensional scaling plots showed a high degree of overlap between cases and controls in both Shetland pony mares and Icelandic horses (Figure 1). Effects of population stratification due to pedigree were therefore limited.

Two imported approved stallion cases were more distant to the other Icelandic horses in our dataset, although the imported Icelandic horses seemed to originate from a similar genetic background (Figure 2). However, imported Icelandic horses (n = 17 cases and n = 2 controls) were removed from the analyses due to less successful matching of Icelandic horses on import status (Table 1 and p = 0.002 for import status). The final Icelandic horse data therefore included 56 cases and 71 controls and were all born in Europe.

### Genome-wide association study

In Shetland pony mares, 13% of variance was explained by all SNP, which is lower than the pedigree-based estimate of heritability of IBH on the liability scale (24%, SE = 6%) in Shetland pony mares in the Netherlands [7]. The 20 non-overlapping windows that explained the largest percentages of genetic variance were located on nine chromosomes (Table 4). The percentage of genetic variance explained by the top 20 associated windows ranged from 0.62 to 0.14% (Table 4) and was highest for the window on chromosome 20 position 35 Mb (Table 4). In 2.7 to 5.3% of iterations of the MCMC (Table 4), the percentage of variance explained by a window exceeded the expected percentage of variance explained (i.e. 0.04%). For each of the top 20 associated windows, the SNP with the highest model frequency is presented in Table 4, including the frequency of the unfavourable allele in cases and controls.

**Table 4:** Windows explaining the largest percentages of insect bite hypersensitivity genetic variance in Shetland pony mares

**Top 20 associated windows**^**a**^					**SNP with highest model frequency within window**				
**ECA**^**b**^	**Position (Mb)**^**c**^	**% of genetic variance explained**^**d**^	**Number of SNP**	**% of iterations where variance explained > 0.04%**^**e**^	**SNP name**	**SNP position (bp)**	**Model frequency**^**f**^	**Allele frequency**^**g**^	
								**Cases**	**Controls**
3	8	0.143	21	2.8	*BIEC2*_*810809*	8,098,240	0.29	0.67	0.54
3	17	0.141	30	3.1	*BIEC2*_*773375*	17,036,655	0.38	0.62	0.46
3	50	0.270	27	3.8	*BIEC2*_*779930*	50,444,836	0.68	0.45	0.29
3	51	0.161	25	3.2	*BIEC2*_*780595*	51,525,184	0.35	0.58	0.43
7	67	0.149	27	3.3	*BIEC2*_*1005528*	67,597,722	0.36	0.67	0.54
7	85	0.171	24	2.9	*BIEC2*_*1010550*	85,800,251	0.29	0.64	0.52
8	63	0.231	22	3.8	*BIEC2*_*1058160*	63,839,900	0.47	0.59	0.44
11	22	0.201	21	3.6	*BIEC2*_*143974*	22,769,190	0.32	0.50	0.35
11	23	0.149	24	3.1	*BIEC2*_*144465*	23,873,176	0.31	0.59	0.45
11	26	0.178	26	3.3	*BIEC2*_*145801*	26,946,633	0.20	0.51	0.39
11	32	0.193	31	4.1	*BIEC2*_*149137*	32,010,755	0.37	0.27	0.13
17	1	0.141	24	2.7	*BIEC2*_*366411*	1,024,001	0.27	0.45	0.31
17	6	0.147	28	2.6	*BIEC2*_*367597*	6,640,619	0.29	0.65	0.51
17	75	0.303	23	4.4	*BIEC2*_*384363*	75,401,514	0.67	0.67	0.52
17	76	0.159	18	2.3	*BIEC2*_*385267*	76,776,877	0.81	0.70	0.55
20	35	0.624	23	5.3	*UKUL3474*	35,643,200	2.03	0.56	0.37
20	41	0.176	21	2.9	*BIEC2*_*532511*	41,520,518	0.84	0.45	0.28
23	14	0.143	25	2.8	*TBIEC2*_*645769*	14,286,784	0.16	0.36	0.27
27	13	0.214	18	3.2	*BIEC2*_*705454*	13,198,799	0.78	0.71	0.54
28	41	0.154	24	3.2	*BIEC2*_*744415*	41,130,845	0.24	0.73	0.61

^a^top 20 1 Mb non-overlapping windows explaining the largest percentages of genetic variance; ^b^*Equus caballus* autosome; ^c^position of the window in Mb pairs, where for instance window position 8 Mb includes SNP located on that particular chromosome between 8 to 9 Mb; ^d^percentage of total genetic variance explained by 1 Mb non-overlapping windows of consecutive SNP based on physical order (build EquCab2.0), averaged across post burn-in iterations, thereby including results from iterations that excluded the window from the model; ^e^percentage of iterations (out of 1799 saved) during which the window captured over 0.04% of genomic variance (i.e. the expected percentage of variance explained by each window in an infinitesimal model); ^f^percentage of iterations where SNP was modelled to have an effect; ^g^frequency of the unfavourable allele.

In Icelandic horses born in Europe, 28% of variance was explained by all SNP, which is equal to the pedigree-based estimate of heritability of IBH on the liability scale (27%, SE = 17%) in Swedish-born Icelandic horses [6]. The 20 windows explaining the largest percentages of genetic variance were located on 14 chromosomes (Table 5). The percentage of genetic variance explained by the top 20 associated windows ranged from 0.66 to 0.14% (Table 5) and was highest for the window on chromosome X position 59 Mb (Table 5). In 2.2 to 7.9% of iterations of the MCMC (Table 5), the percentage of variance explained by a window exceeded the expected percentage of variance explained (i.e. 0.04%). For each of the top 20 associated windows, the SNP with the highest model frequency is presented in Table 5.

**Table 5:** Windows explaining the largest percentages of genetic variance for insect bite hypersensitivity in Icelandic horses

**Top-20 associated windows**^**a**^					**SNP with highest model frequency within window**				
**ECA**^**b**^	**Position (Mb)**^**c**^	**% of genetic variance explained**^**d**^	**Number of SNP**	**% of iterations where variance explained > 0.04%**^**e**^	**SNP name**	**SNP position (bp)**	**Model frequency**^**f**^	**Allele frequency**^**g**^	
								**Cases**	**Controls**
1	7	0.215	25	4.5	*BIEC2*_*2768*	7,759,159	0.94	0.69	0.46
3	35	0.392	18	5.4	*BIEC2*_*776785*	35,897,049	0.45	0.46	0.26
4	24	0.161	17	2.7	*BIEC2*_*855840*	24,611,718	0.32	0.51	0.33
4	43	0.180	18	4.0	*BIEC2*_*861849*	43,590,939	0.51	0.69	0.49
5	26	0.176	20	3.2	*BIEC2*_*898729*	26,364,893	0.52	0.60	0.38
6	6	0.166	43	4.4	*BIEC2*_*937490*	6,127,639	0.69	0.54	0.30
7	55	0.179	24	3.8	*BIEC2*_*1001715*	55,888,542	0.36	0.35	0.18
9	78	0.182	30	3.9	*BIEC2*_*1106244*	78,254,394	0.44	0.43	0.25
11	40	0.266	21	3.5	*BIEC2*_*152809*	40,721,405	1.30	0.67	0.42
15	19	0.162	23	3.7	*BIEC2*_*293503*	19,944,954	0.66	0.55	0.34
15	20	0.211	22	3.1	*BIEC2*_*293623*	20,074,216	1.04	0.68	0.44
15	32	0.142	23	3.2	*BIEC2*_*301468*	32,220,117	0.31	0.59	0.41
15	33	0.381	32	4.9	*BIEC2*-*301721*	33,565,370	2.23	0.70	0.42
18	32	0.179	27	3.9	*BIEC2*_*431445*	32,561,292	0.69	0.54	0.34
19	15	0.186	24	3.5	*BIEC2*_*430270*	15,644,656	0.45	0.54	0.35
19	21	0.151	26	3.3	*BIEC2*_*431289*	21,754,514	0.33	0.75	0.57
20	30	0.162	17	2.2	*BIEC2*_*528135*	30,619,697	0.87	0.78	0.53
23	4	0.159	27	3.4	*BIEC2*_*637804*	4,466,955	0.49	0.74	0.55
X	59	0.658	29	7.9	*BIEC2*_*1126534*	59,703,839	1.04	0.58	0.33
X	60	0.282	29	3.6	*BIEC2*_*1126713*	60,238,370	1.32	0.59	0.33

^a^top 20 1 Mb non-overlapping windows explaining the largest percentages of genetic variance; ^b^*Equus caballus* autosome; ^c^position of the window in Mb pairs, where for instance window position 7 Mb includes SNP located on that particular chromosome between 7 to 8 Mb; ^d^percentage of total genetic variance explained by 1 Mb non-overlapping windows of consecutive SNP based on physical order (build EquCab2.0), averaged across post burn-in iterations, thereby including results from iterations that excluded the window from the model; ^e^percentage of iterations (out of 1799 saved) during which the window captured over 0.04% of genomic variance (i.e. the expected percentage of variance explained by each window in an infinitesimal model); ^f^percentage of iterations where SNP was modelled to have an effect; ^g^frequency of the unfavourable allele.

**Figure 3:**
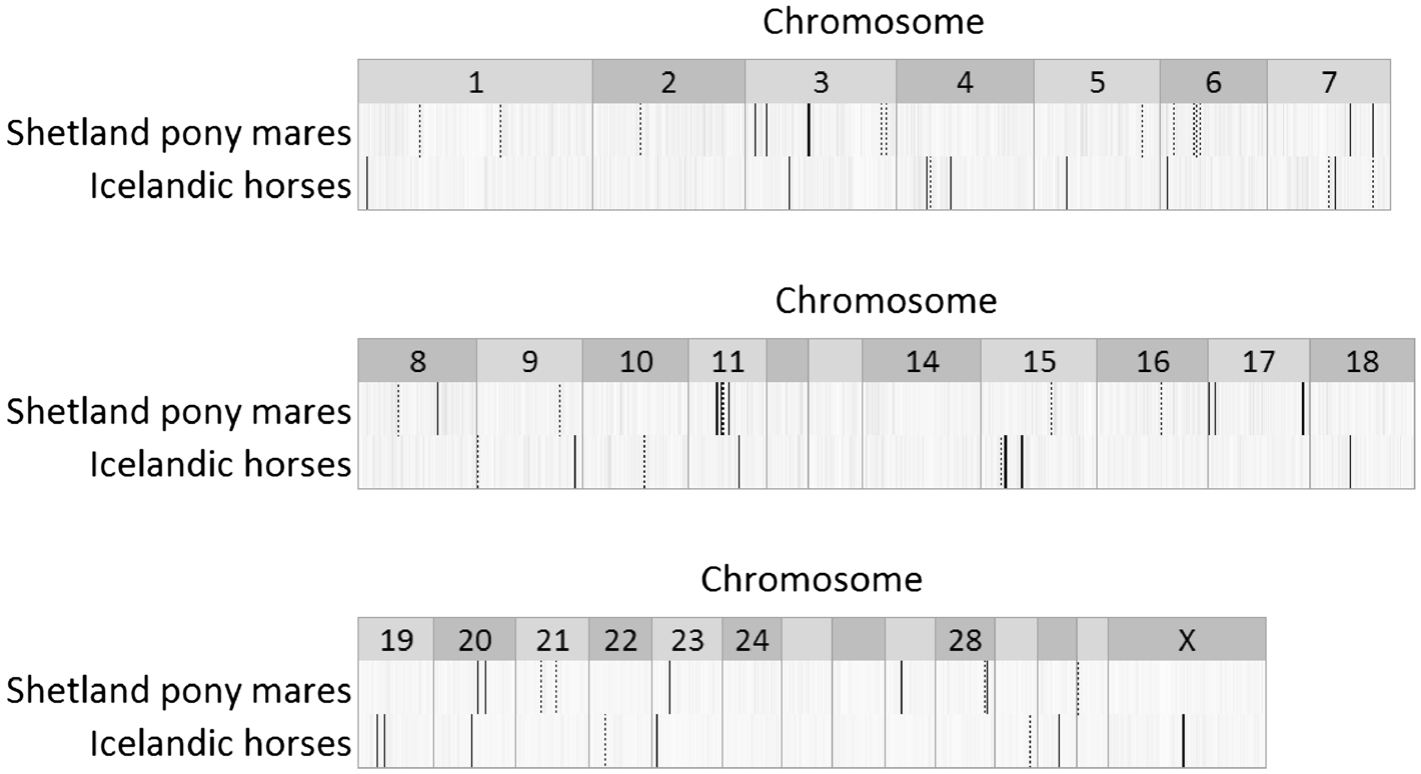
**Heat map comparing the percentage of genetic variance of insect bite hypersensitivity explained by each window in Shetland pony mares and Icelandic horses born in Europe**. Non-overlapping 1 Mb windows are based on the physical order of consecutive SNP across the genome (ECA1 to X; build EquCab2.0); black bars represent windows explaining ≥ 0.14% of genetic variance and dashed black bars represent windows explaining between 0.12 and 0.14% of genetic variance; diminishing grey colour represents a decrease in genetic variance (< 0.12%) explained by windows.

A comparison of associated genomic regions in Shetland pony mares and Icelandic horses (using the percentage of genetic variance explained by 1 Mb non-overlapping windows in the breed-specific GWAS) is depicted in Figure 3. An overlap in the top 20 associated genomic regions (≥ 0.14% of genomic variance explained) was found on chromosomes 3, 7, 11, 20 and 23 (within 5 to 15 Mb), and represent the most promising candidate regions to follow-up on.

## Discussion

The aim of this study was to identify and quantify genomic regions associated with IBH in Shetland pony mares and Icelandic horses in the Netherlands. Breed-specific GWAS were performed and overlapping associated genomic regions (within 15 Mb or less) were identified in both breeds.

### Population stratification analysis

Data were gathered according to a matched case–control design to limit unwanted spurious associations due to population stratification, which might be caused by confounding of ‘the trait of interest’ with pedigree and other relevant (e.g. environmental) effects e.g. [30]. Population stratification due to pedigree was minimized by including paternal half-sib pairs. The multidimensional scaling plots based on breed-specific genomic kinship showed a high degree of overlap between cases and controls (Figure 1). Also, the Bayes method accounts for population stratification due to pedigree by fitting all SNP simultaneously e.g. [31]. In Shetland pony mares, confounding of IBH with relevant effects such as region and withers height category was negligible, since the analysis revealed no significant association between IBH and these effects. In Icelandic horses, importation from Iceland had a significant effect on IBH (p = 0.002). Our results showed (Figure 2) that differences in genetic background between imported Icelandic horses and Icelandic horses born in Europe were limited, which agrees with Broström et al. [32] and Andersson et al. [10]. However, *Culicoides* spp. is absent in Iceland and consequently IBH is not observed e.g. [33]. Increased environmental pressure after export and lack of exposure to *Culicoides* spp. before export are suggested to result in increased incidence and more severe cases after export e.g. [32,34]. The insect bite hypersensitivity statuses of imported Icelandic horses and Icelandic horses born in Europe may not represent the exact same phenotype. The final Icelandic horse data, therefore, only included horses born in Europe.

### Single SNP and multi-locus models

Schurink et al. [14] published genomic regions associated with IBH in 188 Shetland pony mares using 50 k SNP genotypes. In our study, several similar associated genomic regions within 1 Mb distance were identified in Shetland pony mares on chromosomes 3, 11, 20 and 27. However, Schurink et al. [14] used logistic regression fitting single SNP effects, while our Bayes-C method fitted all SNP simultaneously. Mucha et al. [35] concluded that estimated variances of identified QTL were not overestimated when all SNP were fitted simultaneously, since the variance explained will be distributed across all SNP in high LD with the QTL and therefore cannot exceed the total variance (in contrast to single SNP analysis). Indeed, Sahana et al. [36] compared various association mapping methods and showed that a Bayesian variable selection model that fitted all SNP simultaneously performed best overall. The Bayesian variable selection model using the posterior probability of a QTL in 1 cM overlapping regions to identify associated genomic regions had the highest power to map small QTL (i.e. explaining 2% of genetic variance) and most precise estimates of QTL location. However, a mixed model analysis fitting random additive genetic effects and testing single SNP performed almost as well, although it was computationally more demanding and multiple testing correction was needed. Like in Sahana et al. [36], analysis of the Shetland pony mare data using logistic regression with single SNP effects, as in Schurink et al. [14], was computationally much more demanding than the Bayesian variable selection method used here and ignored dependencies between SNP. Although several similar associated genomic regions were identified using these two methods, Bayesian variable selection model using posterior probabilities of genomic regions is preferred as it is computationally less demanding, it does not require correction for multiple testing and it accounts for population stratification due to pedigree by fitting all SNP simultaneously.

### Non-overlapping window approach

The window approach takes LD between SNP into account and is therefore a better criterion for QTL identification than posterior probabilities of single SNP [23,36]. However, optimal choice of the size of a window is not clear, as a specific window may contain more than one QTL or a QTL may be spread over more than one window [27]. For example, after merging windows at 75 and 76 Mb on chromosome 17 in Shetland pony mares and performing another GWAS, the percentage of variance explained by this 2 Mb genomic region was 0.426, which roughly equals the sum of genetic variance explained by the two separate 1 Mb windows (Table 4). Because these 1 Mb windows were consecutive, the percentage of variance explained by the 2 Mb windows might be considered as total QTL variance (if indeed the two consecutive 1 Mb windows represent the same QTL), whereas the percentage of variance explained by each 1 Mb might each represent a proportion of QTL variance. However, the true QTL position might not be contained in the window with strongest association. Precision of QTL mapping depends on several factors, such as the method of analysis, marker density, sample size and variance explained by the QTL [37]. In a simulated data set of binary phenotypes and SNP genotypes by Mucha et al. [35], the mean distance of estimates from true QTL positions ranged from 0.30 to 0.77 Mb, depending on the method of analysis used. However, the SNP density simulated by Mucha et al. [35] was higher than in our study. Because LD can differ between genomic regions e.g. [38,39], LD within a genomic region could be used to determine the optimal size of a window in a given region, although further research is needed to determine the relationship between LD structure and optimal window size.

### Genome-wide association study

Associated genomic regions identified in both breeds (Figure 3) suggest interesting candidate genomic regions to follow-up on. A simultaneous GWAS of both breeds is expected to increase power to detect associations, as more data would be included. However, GWAS across breeds will be less likely to detect SNP that are in LD with QTL in only one breed and will be more likely to detect SNP in LD with QTL across both breeds, provided LD phase is conserved across breeds e.g. [40,41]. To meet these requirements, SNP and QTL need to be physically close or, ideally, represent the actual mutation (which is unlikely). De Roos et al. [41] concluded that roughly 50 000 SNP are required to have sufficient LD (i.e. ≥ 0.20) for genomic selection within a dairy cattle breed but that 300 000 SNP are required to find SNP that are in LD with the QTL across breeds. Persistency of LD phase extended less than 10 kb between bovine breeds that diverged hundreds of generations ago [41]. The consistency of LD phase between Shetland ponies and Icelandic horses was not investigated. Shetland ponies and Icelandic horses did cluster together in the phylogenetic analysis of van de Goor et al. [42], which used equine short tandem repeat loci. However, divergence of the breeds occurred many generations ago, thus LD from the ancestral population is expected to have been broken down [43]. Also, the current equine SNP density results in insufficient LD (roughly 0.3 [44]) to expect to find SNP that are in LD with QTL across breeds.

### Candidate genes

Research on IBH using the candidate gene approach or GWAS in horses has been limited. Using a candidate gene approach, Andersson et al. [45] concluded that *SPINK5* (serine peptidase inhibitor, Kazal type 5) on ECA14 was not associated with IBH in Swedish-born Icelandic horses. In our study, no genomic regions associated with IBH were found on ECA14. Hořin et al. [46] investigated polymorphisms in various immune response related genes to identify associations with *R*. *equi* and *L*. *intracellularis* that cause respectively lung and gastrointestinal infections in horses. Several polymorphisms were significantly associated with these infections, including microsatellite locus HMS01 on ECA15. Marti et al. [47] in [48] concluded that locus HMS01 is associated with IBH. In our study, genomic regions associated with IBH were identified on ECA15 but only in Icelandic horses. Various *IL1* (interleukin 1) related genes are located in or around these regions.

We anticipated a common genetic background of IBH across breeds, although breed-specific genetic influences on IBH cannot be excluded. However, SNP densities within genomic regions could differ between Shetland pony mares and Icelandic horses due to breed-specific edits based on MAF and call-rate. Also, LD between SNP and QTL might be present in one breed but absent in the other e.g. [41], thereby impeding validation of QTL across breeds. Associated genomic regions identified in both Shetland pony mares and Icelandic horses were considered most interesting to follow-up on and were found on ECA3, 7, 11, 20 and 23 (Figure 3, Tables 4 and 5). However, positional candidate genes adjacent to associated genomic regions were identified only for the genomic region on ECA20. No other candidate gene with known function in immunology or allergy was identified in or adjacent to across-breed associated genomic regions. The equine lymphocyte antigen (ELA) class II region is located on ECA20 (spanning 32 and 33 Mb) between the associated genomic regions identified in the Shetland pony mares and Icelandic horses (Tables 4 and 5, Figure 3). ELA, or equine major histocompatibility complex, evokes an immune response by recognizing many foreign molecules [13]. Both serological [11,12] and genomic research [10] have identified an association between ELA class II antigens and IBH. Andersson et al. [10] concluded that the same allele at an ELA locus is associated with IBH in two distinct horse breeds and homozygosity across the ELA region increased IBH sensitivity. An association with IBH on ECA20 was also found by Schurink et al. [14], although the identified region was approximately 8 Mb away from the ELA class II region. However, coverage within the region was poor for the Illumina® EquineSNP50 Genotyping BeadChip (Illumina Inc.) used by Schurink et al. [14], but improved in the current equine HD chip. Associated genomic regions on ECA20 that were identified in the Shetland pony mares and Icelandic horses were within 2 Mb from the ELA class II region, which is reasonably close to confirm the impact of ELA class II region on IBH.

### Conclusions and implications

The genome-wide association study performed here identified several genomic regions associated with IBH in both Shetland pony mares and Icelandic horses. On ECA20, associated genomic regions were identified in both breeds that were within 2 Mb from the equine lymphocyte antigen class II region containing candidate genes. Knowledge on genes associated with IBH will contribute to our understanding of its biology, enabling more efficient therapy, prevention and selection in order to decrease IBH prevalence. Sequencing candidate genes within the equine lymphocyte antigen class II region might identify the functional mutation. Selection on functional mutations, i.e. direct markers, is more effective than indirect markers (i.e. LD and linkage equilibrium markers) [49]. However, genetic gain for marker-assisted selection using only a small number of significant markers to trace a limited number of QTL (although often with larger effects) is likely to be small because a large number of QTL are expected to explain genetic variation in complex traits e.g. [16]. In genomic selection, dense genome-wide markers are used to estimate genomic breeding values based on marker effects across the entire genome. Marker density is assumed to be sufficient so that each QTL is in LD with at least one marker or with a set of markers. Therefore, genomic selection could potentially capture the total genetic variance for a complex trait e.g. [16]. Possibilities for genomic selection on IBH in horse populations or even across horse populations and corresponding implications must be investigated before implementation is considered.

## Competing interests

The authors declare that they have no competing interests.

## Authors’ contributions

AS acquired the data, carried out the genome-wide association study and drafted the manuscript. BJD, KF and JAMA made substantial contributions to the acquisition of the data, interpretation of results and helped to draft and revise the manuscript. AW, DJG and JCMD made substantial contribution to analysis and interpretation of results and helped to draft and revise the manuscript. DJG and JCMD contributed to the development of GenSel software ( http://bigs.ansci.iastate.edu/). All authors read and approved the final manuscript.
